# Prevalence, Concordance, and Heritability of Vitreomacular Interface Abnormalities in a Twin Study

**DOI:** 10.1167/iovs.64.10.9

**Published:** 2023-07-10

**Authors:** Zakariya A. Jarrar, Abdus Samad Ansari, Katie M. Williams, Dominic S. Wong, Pirro G. Hysi, Omar A. Mahroo, Christopher J. Hammond

**Affiliations:** 1 Section of Ophthalmology, King's College London, London, England, United Kingdom; 2 Department of Twin Research and Genetic Epidemiology, King's College London, London, England, United Kingdom; 3 Institute of Ophthalmology, University College London, and Moorfields Eye Hospital, London, London, England, United Kingdom

**Keywords:** vitreoretinal pathologies, epidemiology, genetic diseases, retina

## Abstract

**Purpose:**

The relative importance of genetic factors in common vitreomacular interface (VMI) abnormalities is unknown. The aim of this classical twin study is to determine the prevalence case wise concordance between monozygotic and dizygotic twin pairs, and heritability of common VMI abnormalities, including epiretinal membrane (ERM), posterior vitreous detachment (PVD), vitreomacular adhesion (VMA), vitreomacular traction (VMT), lamellar macular holes (LMHs), and full-thickness macular holes (FTMHs).

**Methods:**

This is a single-center, cross-sectional classical twin study of 3406 TwinsUK participants over the age of 40 years who underwent spectral domain macular optical coherence tomography (SD-OCT) scans which were graded for signs of VMI abnormalities. Case wise concordance was calculated and the heritability of each VMI abnormality was estimated using OpenMx structural equation modeling.

**Results:**

In this population (mean age = 62.0 years [SD = 10.4 years], range = 40–89 years) the overall prevalence of ERM was 15.6% (95% confidence interval [CI] = 14.4–16.9) and increased with age, posterior vitreous detachment affected 21.3% (20.0–22.7), and VMA was diagnosed in 11.8% (10.8–13.0). Monozygotic twins were more concordant for all traits than dizygotic twins, and age, spherical equivalent refraction (SER), and lens status-adjusted heritability was estimated at 38.9% (95% CI = 33.6–52.8) for ERM, 53.2% (95% CI = 41.8–63.2) for PVD, and 48.1% (95% CI = 33.6–58) for VMA.

**Conclusions:**

Common VMI abnormalities are heritable and therefore have an underlying genetic component. Given the sight-threatening potential of VMI abnormalities, further genetic studies, such as genomewide association studies, would be useful to identify genes and pathways implicated in their pathogenesis.

Vitreomacular interface (VMI) abnormalities encompass numerous pathologies including epiretinal membrane (ERM), full-thickness macular holes (FTMHs), and lamellar macular holes (LMHs), as well as vitreomacular adhesion (VMA), and vitreomacular traction (VMT). VMI abnormalities can cause central vision problems, including reduced visual acuity and metamorphopsia. Symptom severity can vary greatly at presentation. The prevalence of these abnormalities has varied between large population-based studies,^1^ largely due to differences in grading and imaging devices used. Traditionally, color fundus photography (CFP) has been used in population-based studies to classify VMI abnormalities and prevalence estimates vary greatly, for example, ERM prevalence estimates using CFP vary from 3.4 to 28.9%.^2^^–^^11^ Since the widespread introduction of spectral domain optical coherence tomography (SD-OCT), population-based studies^9^^,^^12^^–^^17^ have used it to report VMI abnormalities given it is more sensitive than CFP, particularly in earlier stages.^18^ However, despite the introduction of SD-OCT, a large amount of variation still exists between studies.

Although risk factors for certain VMI abnormalities have been documented, such as advancing age for ERM^13^^,^^14^^,^^17^ and PVD,^19^ many cases are labeled idiopathic and little is known about their etiology, and whether genetic factors contribute. Secondary causes of ERM include intraocular inflammation, diabetes, and retinal vascular occlusion. Studying twins may help understand the relative importance of genetic variation in etiology. To our knowledge, no previous studies have reported the heritability or concordance of these traits. Therefore, this study aims to report the prevalence, case wise concordance, and heritability of VMI abnormalities from the TwinsUK cohort.

## Methods

Established in 1992 and now with over 15,000 twins, the TwinsUK cohort is the largest twin registry in the United Kingdom and one of the most deeply phenotyped adult twin cohorts studying common age-related diseases. Details of the cohort have been described previously.^20^ The TwinsUK cohort is predominantly female subjects, partly due to its establishment to examine osteoporosis and other rheumatological diseases in the early 1990s, and due to a female volunteer bias common to most twin cohorts.

### Eye Examination

Since 2014, TwinsUK participants have had non-mydriatic disc and macular CFP and SD-OCT scans (Optovue iVue 100; Optovue, Freemont, CA, USA), performed by a trained technician in a dark room. The 3D Macula protocol was selected which contains 128 B-scans from a 6 × 6 mm fovea-centered square. Where the technician deemed the image quality to be inadequate, a repeat scan was taken. Images where the central foveal scan was of good quality and gradable were included in this study. Where longitudinal data were available, the latest SD-OCT scans for a given participant were selected. One trained ophthalmologist (author Z.J.) masked to participant zygosity retrieved and graded macular SD-OCT scans for VMIs according to the European Eye Epidemiology (E3) Consortium classification of macular disease for epidemiological studies.^21^

VMI abnormalities in this classification system are based on the International Vitreomacular Traction Study Group (IVTS) definitions, which have previously been described.^22^

PVD diagnosed from macular SD-OCT relied on the posterior hyaloid being visualized, either being totally, or partially detached from the inner retinal surface. Where the posterior hyaloid face was not clearly seen on macular SD-OCT scans, the eye was graded as not having PVD. For the analyses, a case was defined as the presence of a given VMI abnormality in either eye. Given that VMI abnormalities are rare in the population under the age of 40 years, only participants aged 40 years and above were included in this study. A subset of 50 SD-OCT images were then selected for independent grading by a second ophthalmologist (author A.S.A.) and interobserver percentage agreement and Cohen's kappa were calculated for each VMI abnormality. Where graders differed, the disagreements were resolved to reach a final consensus.

Questionnaire data on ocular pathology were collected from study participants. Given the rarity of self-reported possible secondary VMI causes we did not initially exclude these subjects as the aim was to report heritability of any VMI, regardless of known or unknown cause.

### Zygosity

Twin zygosity was ascertained using available genotyping array data, and the “peas in a pod” questionnaire, which has been shown to be a cost-effective, accurate proxy for determining zygosity.^23^ In the event of uncertain zygosity, confirmation was obtained with genetic testing using The AmpFl STR Profiler kit (PE Applied Biosystems, Foster City, CA, USA).

### Ethics

Ethical approval was granted, and participants’ written consent was obtained prior to data collection. The research adhered to tenets of the Declaration of Helsinki.

### Analytical Approach

Determining the effect of genes and environment in family studies can be difficult due to the shared effects of each between family members. However, twin pairs are assumed to have the same early shared environment, so greater concordance of a phenotype within monozygotic (MZ) twins than dizygotic (DZ) twins is attributed to their greater genetic sharing (100% cf approximately 50%) and is evidence of a genetic role in etiology.^24^ The concordance of a trait in twins is the probability that both twins will have a certain trait given that one twin has the trait. Case wise concordance was calculated for MZ and DZ twin pairs using 2C/(2C + D), where C = number of twin pairs concordant for, and D = number of twin pairs discordant for a given phenotype.

Liability threshold modeling in twin studies using binary data relies on the assumption that the likelihood of developing a disease is normally distributed and that where disease is present, a certain threshold has been met.^25^ Maximum likelihood structural equation modeling techniques were used to determine the relative contributions of additive (A) genetic effects, dominant (D) genetic effects or common environmental effects (C), and unique environmental effects (E) to the variation of all phenotypes, and adjusted for age, spherical equivalent refraction (SER), and lens status, using the OpenMx (http://openmx.psyc.virginia.edu/) package in R (RStudio, PBC, Boston, MA, USA).^26^

The significance of each component of the model (A, C, D, and E) was assessed, by omitting each component in submodels and determining the deterioration in the model fit after each omission. If after a component's removal, there is a significant change in the model fit, the omitted component is deemed significant and retained in the original model. The most parsimonious model is determined using the lowest Akaike Information Criterion (AIC), which describes the model of best fit with the fewest number of components. In the final model, the heritability estimate is the contribution of genetic factors to trait variance, either “A” or “A + D.”

## Results

A total of 7837 macular SD-OCT images from 3411 twin participants aged over 40 years and over were collected. After image quality control, 6733 macular SD-OCT scans with a clear, gradable foveal image from 3406 twin participants (1574 complete twin pairs, 941 MZ, and 633 DZ) were graded for signs of VMI abnormalities and included in the analysis. Interobserver agreement in a random sample was high, with percentage agreement of VMI abnormalities graded on SD-OCT ranging from 88% to 100% and Cohen's kappa results indicating substantial to perfect agreement (Supplementary Table S1). Self-reported prevalence estimates were: 1.4% retinal detachment, 0.9% diabetic retinopathy, 0.3% uveitis, 0.3% retinal tear, 0.06% retinal vein occlusion, and 0.03% intraocular tumor. There were 8.3% of participants who self-reported pseudophakia.

**Figure 1.**
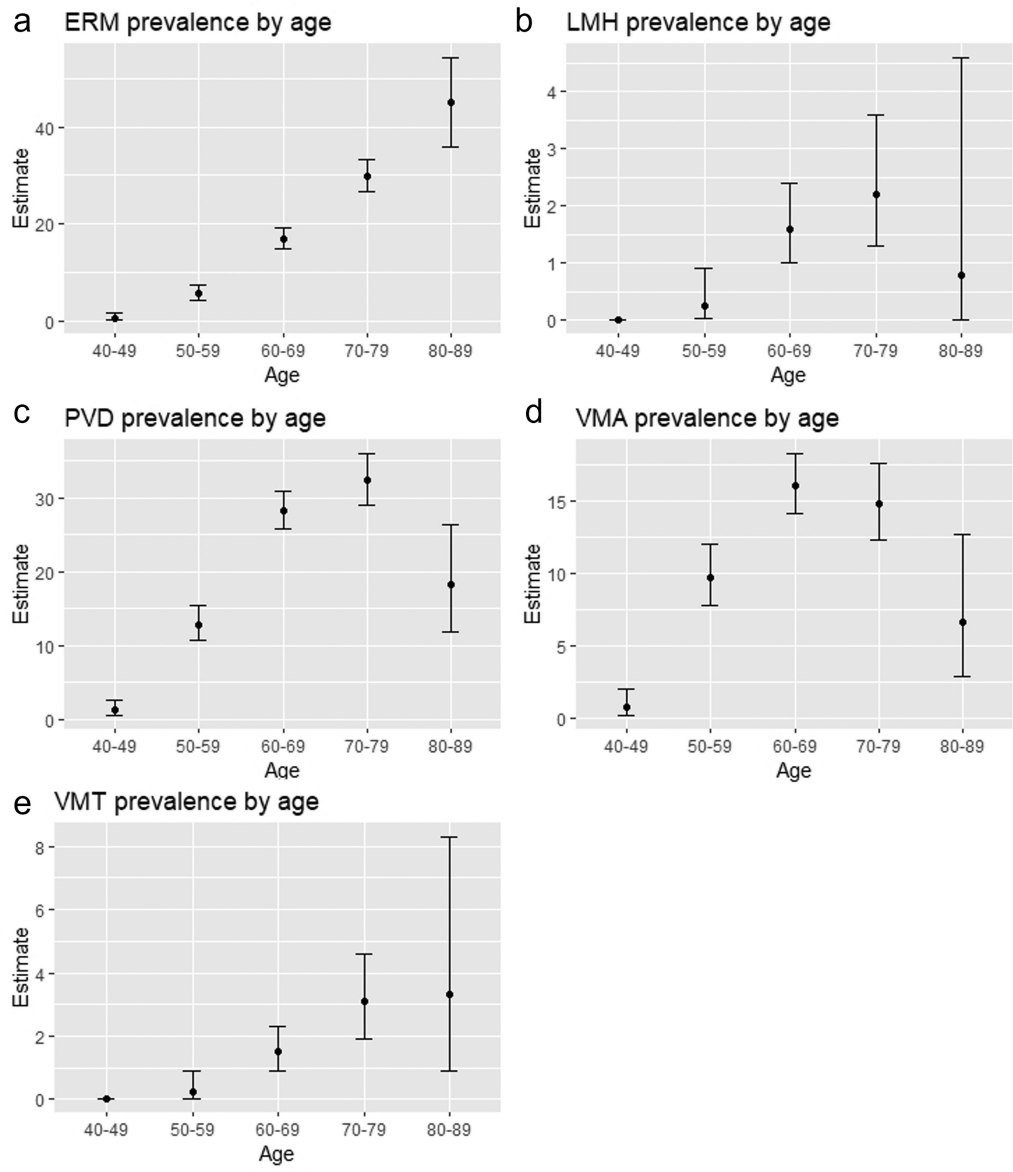
(**a–e**) Prevalence estimates of vitreomacular interface abnormalities and 95% confidence intervals stratified by age (years). ERM, epiretinal membrane; LMH, lamellar macular hole; PVD, posterior vitreous detachment; VMA, vitreomacular adhesion; VMT, vitreomacular traction.

The mean age of the participants was 62.0 years (SD = 10.4 years), range 40 to 89 years (median = 63 years), 87.6% were female, and 95% were White Caucasians. The proportions of participants with the following in at least one eye were as follows: ERM 15.6% (95% CI = 14.4–16.9), FTMH 0.03% (95% CI = 0.0–0.2), LMH 1.2% (95% = CI 0.8–1.6), PVD 21.3% (95% CI = 20.0–22.7), VMA 11.8% (95% CI = 10.8–13.0), and VMT 1.4% (95% CI = 1.0–1.8). ERM was strongly age-related with prevalence estimates ranging from 0.4% (95% CI = 0.05–1.5) in 40 to 49-year-olds to 45% (95% CI = 35.9–54.3) in 80 to 89-year-olds, *P* < 0.0001. Figures 1A to E and Table 1 show the age-stratified prevalence estimates and 95% CIs of VMI abnormalities.

**Table 1.** Prevalence Estimate, % (95% Confidence Intervals), of VMI Abnormalities Stratified by Age

Age, y Trait	40–49 (*n* = 481)	50–59 (*n* = 813)	60–69 (*n* = 1270)	70–79 (*n* = 722)	80–89 (*n* = 120)	All (*n* = 3406)
ERM	0.4% (0.05–1.5)	5.5 (4.1–7.3)	16.9 (14.9–19.1)	29.8 (26.5–33.3)	45 (35.9–54.3)	15.6 (14.4–16.9)
FTMH	—		—	—	0.8 (0.02–4.6)	0.03 (0–0.2)
LMH	—	0.3(0.03–0.9)	1.6 (1–2.4)	2.2 (1.3–3.6)	0.8 (0.02–4.6)	1.2 (0.8–1.6)
PVD	1.3% (0.46–2.7)	12.9 (10.7–15.4)	28.3 (25.8–30.8)	32.4 (29–36)	18.3 (11.9–26.4)	21.3 (20.0–22.7)
VMA	0.8 (0.2–2.1)	9.7 (7.8–12)	16.1 (14.2–18.3)	14.8 (12.3–17.6)	6.7 (2.9–12.7)	11.8 (10.8–13.0)
VMT	—	0.25 (0.03–0.9)	1.5 (0.9–2.3)	3.1 (1.9–4.6)	3.3 (0.9–8.3)	1.4 (1.0–1.8)

ERM, epiretinal membrane; FTMH, full thickness macular hole; LMH, lamellar macular hole; PVD, posterior vitreous detachment; VMA, vitreomacular adhesion; VMT, vitreomacular traction.

The prevalence estimates for male and female subjects, respectively, for the following traits were: ERM = 15.4% (95% CI = 12.1–19.2) and 15.6% (95% CI = 14.3–16.9) *P* = 0.46, FTMH = 0% and 0.03% (95% CI = 0.0–0.2), LMH = 0.5% (95% CI = 0.1–1.7) and 1.2% (95% CI = 0.9–1.7) *P* = 0.1, PVD = 13.5% (95% CI = 10.4–17.1) and 22.4% (95% CI = 20.9–23.9) *P* < 0.001, VMA = 7.1% (95% CI = 4.9–10.0) and 12.5% (95% CI = 11.3–13.7) *P* < 0.001, and VMT = 0.6% (95% CI = 0.1–1.6) and 1.3% (95% CI = 1.0–1.8) *P* = 0.11, respectively.

Male and female subjects were similar ages (mean age = 62.1 years [10.7], and 61.9 years [10.3], respectively, *P* = 0.36). MZ twin pairs were slightly younger on average than DZ twin pairs (mean age = 60.8 years [10.8] vs. 62.9 years [9.3], *P* < 0.01, respectively), which might have contributed to a difference in PVD prevalence (MZ prevalence = 19.6% [95% CI = 17.8–21.5], and DZ prevalence = 23.5% [95% CI = 21.2–26.0], *P* = 0.01). There were no significant differences in the prevalence estimates of ERM, FTMH, LMH, and VMA or between MZ and DZ twin pairs.

The prevalence of ERM in the 8.3% of pseudophakic participants was 40.6% compared to 13.3% in phakic subjects, *P* < 0.0001; for PVD prevalences were 22.8% vs. 21.8%, *P* = 0.70, and for VMA 8.2% vs. 12.5%, *P* = 0.04. Overall, there were no significant differences in SER between cases and controls for the VRI abnormalities (ERM vs. no ERM = 0.11 [SD = 2.61] vs. 0.07 [SD = 2.55], *P* = 0.41; PVD vs. no PVD = 0.72 [SD = 6.7] vs. 0.29 [SD = 6.5], *P* = 0.09; VMA vs. no VMA = 0.69 [SD = 5.65] vs. 0.29 [SD = 6.07], *P* = 0.10).

### Case Wise Concordance

**Figure 2.**
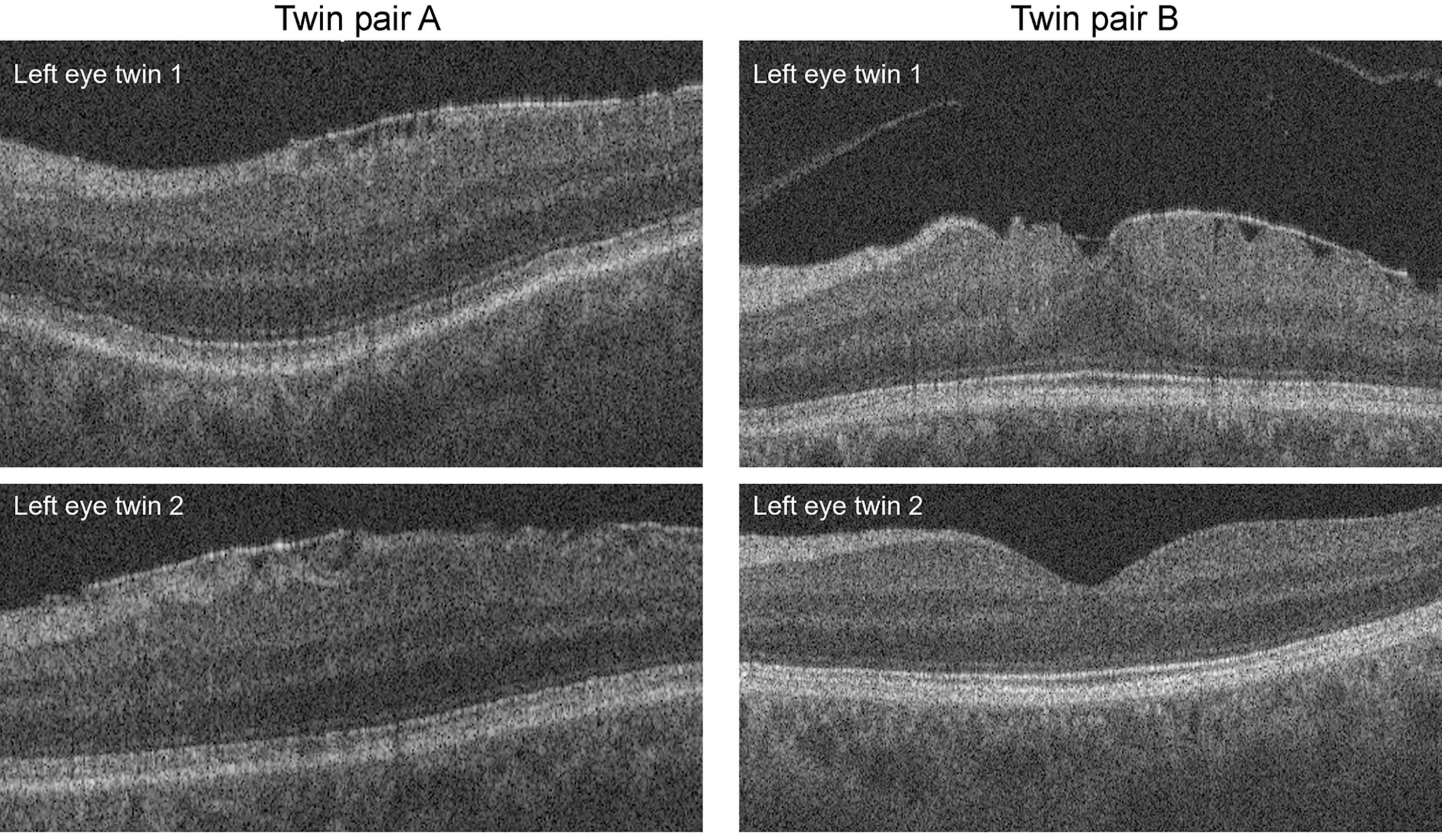
Macular OCT scans from two female monozygotic twin pairs (**A,** **B**). The scans on the top are from an 89-year-old twin pair and show the concordance of epiretinal membrane (ERM) in both twins’ left eyes. The images on the bottom are from a 69-year-old twin pair and show the discordance for epiretinal membrane in both twins’ left eyes.

Case wise concordance calculations for MZ and DZ twins were only performed for ERM, PVD, and ERM, given low numbers of affected individuals for other VMI abnormalities (Table 2). Concordances for ERM, PVD, and VMA within MZ and DZ twins were 0.47 and 0.27, 0.48 and 0.38, and 0.36 and 0.22, respectively. Figure 2 shows an example of macular SD-OCT scans from 2 MZ twin pairs; one concordant and one discordant pair for ERM.

**Table 2.** Case Wise Concordances of VMI Abnormalities in MZ and DZ Twins

VMI Abnormality	No. of Twin Pairs Concordant for Trait	No. of Twin Pairs Discordant for Trait	Concordance	Risk Ratio MZ/DZ
**ERM**				
MZ	83	188	0.47	1.74
DZ	33	180	0.27	
**FTMH**				
MZ	0	0	−	−
DZ	0	1	−	
**LMH**				
MZ	1	21	−	−
DZ	1	15	−	
**PVD**				
MZ	88	193	0.48	1.26
DZ	56	186	0.38	
**VMA**				
MZ	45	158	0.36	1.64
DZ	23	1623	0.22	
**VMT**				−
MZ	1	21	−	
DZ	0	18	−	

DZ, dizygotic twin pairs; ERM, epiretinal membrane; FTMH, full thickness macular hole; LMH, lamellar macular hole; MZ, monozygotic twin pairs; PVD, posterior vitreous detachment; VMA, vitreomacular adhesion; VMT, vitreomacular traction.

Given the pathology of idiopathic ERM is likely to be different to secondary ERM, we examined the concordances after removing participants with self-reported secondary causes of VRI abnormalities, including retinal detachment or tear, uveitis, diabetic retinopathy, and intraocular tumor (*n* = 101). A further 26 participants were excluded for signs of late AMD on SD-OCT and 88 due to intraretinal fluid. ERM concordance was 0.44 for MZ pairs c.f. 0.24 for DZ pairs (similar to all-cases concordances 0.47 and 0.27). PVD and VMA concordances were also similar.

### Heritability of VMI Abnormalities

Heritability models adjusted for age, pseudophakia, and SER combining additive genetic and unique environmental effects provided the best model fit for all traits, resulting in heritability estimates for ERM, any PVD, and VMA of 38.9% (95% CI = 33.6–52.8), 53.2% (95% CI = 41.8–63.2), and 48.1% (95% CI = 33.6–58), respectively.

## Discussion

This study has shown that common VMI abnormalities are heritable and, to our knowledge, is the first classical twin study to do so. Based on this work, future genetic studies, such as genomewide association studies (GWAS) may be able to elucidate mechanisms by which VMI abnormalities form and aid the development of future potential treatments.

### Concordance and Heritability

This study found the heritability of ERM to be 38.9% (33.6–52.8) with individual environmental effects (which includes grading error and stochastic, or random, effects) explaining the remaining variance. The concordance of ERM for MZ and DZ twins was 0.47 and 0.27, respectively (i.e. if one twin in an MZ twin pair has ERM, the probability their co-twin having ERM is 0.47, and if one twin in a DZ twin pair has ERM, the probability their co-twin having ERM is 0.27). The heritability and higher concordance of ERM in MZ than DZ twins suggests a role for genetic factors in the development of ERM.

After removing self-reported and SD-OCT derived secondary causes of VMI, the concordances of ERM, PVD, and VMA changed very little and, in fact, the risk ratio for MZ/DZ increased for each VMI abnormality: ERM = 1.89, PVD = 1.47 and VMA = 1.8.

To date, few studies with small participant numbers have explored the genetics of ERM and these have predominantly focused on gene expression in vitreous samples of eyes undergoing ERM surgery.^27^^,^^28^ From eyes undergoing surgery for idiopathic epiretinal membrane, genes involved in angiogenesis and wound healing, such as *IL6*, *TGFB2*, and *VEGFA* among others have been highlighted, although these changes may reflect expression changes consequent to rather than causal of ERMs.^27^ GWASs with large sample sizes are needed to identify genes implicated in the development of ERM. Although age, posterior vitreous detachment, and ERM in the fellow eye have been identified as risk factors for idiopathic ERM development, few environmental risk factors have been reported or replicated. A pooled odds ratio of seven population-based cohorts found smoking to be protective for ERM, OR = 0.67 (0.58–0.78), although the results should be interpreted with caution due to possible survivor bias.^1^

The heritability of VMA and PVD was estimated at 48.1% (95% CI = 33.6–58), and 53.2% (95% CI = 41.8–63.2), respectively. The concordance of VMA and PVD for MZ and DZ twins was 0.36 and 0.22, and 0.48 and 0.38, respectively, which suggests that genetic factors may influence adherence of the vitreous at the macula. Indeed, mutations in laminin alpha-5, a gene encoding extracellular matrix glycoproteins implicated in cell adhesion, have been associated with early posterior vitreous detachment independent of age in a family study.^29^ This study did not measure the area of VMA but used the European Eye Epidemiology SD-OCT classification of broad VMA >1500 µm or focal VMA <1500 µm. We, like others,^30^ found the 2 eyes of the 112 individuals with bilateral VMA were highly correlated, 99.1% shared the same width classification. The majority of twins (88%) had focal VMA, and 49 of 68 twin pairs concordant for VMA shared the same focal classification, with 19 pairs having one individual with narrow and one with broad attachment width. No pairs were concordant for broad width. This study is underpowered to examine heritability of the subtypes of VMA.

Our study was underpowered to calculate concordances and heritability for FTMH, LMH, and VMT. However, family studies report a higher occurrence of macular hole in family members than unrelated controls, suggesting a genetic component in its development,^31^^,^^32^ although unlike twin studies, these types of studies cannot easily control for shared family environmental factors.

### Prevalence

Our overall ERM prevalence estimate in the population over 40 years was 15.6% (14.4–16.9). The reported prevalence estimates for ERM diagnosed from SD-OCT in published literature vary greatly from 4.7% to 52.8%^9^^,^^12^^–^^14^^,^^16^^,^^17^ and our estimate lies within this range. The varying ERM prevalence estimates between studies are likely due to differing classifications and definitions of ERM, devices used, and age as well as other characteristics of population demographics.

Unlike other VMI abnormalities defined by the IVTS group, there is no international consensus on the grading of the severity of ERM from SD-OCT. Some studies have devised their own classifications,^17^^,^^33^^,^^34^ but, in this study, an ERM was defined by the presence of a hyper-reflective inner retinal band in either eye, irrespective of stage or severity (as it was in the NICOLA study, which is closest geographically and ethnically to the TwinsUK cohort).

Different OCT devices and imaging protocols may provide varying image quality which may influence diagnosis of VMI abnormalities and therefore prevalence estimates. For example, in the Blue Mountains Eye Study, a time-domain OCT with lower resolution used in the earlier phases of the study was less accurate for detecting ERM than SD-OCT.^12^

Variation in prevalence estimates also exists in CFP grading of ERM from 3.4% to 28.9%.^1^ Whereas SD-OCT is more sensitive than CFP for detecting ERM, particularly at earlier stages,^18^ significant differences in reported prevalence exist between studies and perhaps an international consensus on ERM classification could help ensure better comparability between study estimates.

Like other studies, we found ERM was associated with increasing age, with a prevalence estimate of 45% (95% CI = 35.9-54.3) over the age of 80 years, similar to prevalence estimates found in other older cohorts of Alienor (52.8%), mean age of 79.4 ± 4.2 years,^15^ Montrachet (38.9%), mean age of 82.3 ± 3.8 years,^17^ and Beaver Dam Eye Study (34.1%), mean age of 74.1 ± 7.1 years.^13^

Individual studies differ as to whether sex is associated with ERM. For example, the NICOLA study found the odds of ERM diagnosed on SD-OCT decreased with female sex, odds ratio (OR) = 0.69 (95% CI = 0.52-0.91, *P* = 0.01).^14^ However, a large meta-analysis of risk factors for ERM diagnosed from CFP found the female sex to be a risk factor for ERM OR = 1.34 (95% CI = 1.17–1.53).^1^ Our study found no significant sex difference in the prevalence of ERM (male subjects = 15.4% [95% CI = 12.1–19.2], female subjects = 15.9% [95% CI = 11.8–14.1], *P* = 0.68).

It is well established that PVD is age-related and associated with high myopia.^35^ Subjects in our study with PVD were older than those without (mean age = 66.3 [SD = 6.9] vs. 60.3 [SD = 10.6], *P* < 0.001, respectively). The prevalence of high myopia (<−6.00 diopters [D]) in MZ and DZ twin pairs was 3.2% (95% CI = 2.3–4.2%) and 2.5% (95% CI = 1.6–3.7%) respectively, *P* = 0.16. The prevalence of VRI abnormalities in participants with high myopia were slightly higher than overall: ERM = 18.6% (95% CI = 10.3–29.7), PVD = 10.6% (95% CI = 4.4–20.6), and VMA = 5.6% (95% CI = 1.6–13.8). There was no significant difference in mean SER between those with and without PVD. We found a higher prevalence of PVD in female than male subjects, 22.4% (95% CI = 20.9–23.9) vs. 13.5% (95% CI = 10.4–17.1), *P* < 0.001, respectively. A previous study found PVD progressed at a significantly faster rate in female eyes than male eyes at age 60 years,^19^ reporting that changes at the vitreomacular interface occur earlier in women. There are differing results from large population-based studies examining sex differences in myopia. For example, a 2015 UK Biobank study of 107,452 participants (mean age = 56.6 years) reported a higher OR of any myopia and also hypermetropia among women.^36^ However, a European meta-analysis of refractive error on 61,946 participants (median age = 62 years) found no significant difference in myopia rates between the sexes.^37^ In this twin study, there was no significant difference in the prevalence of high myopia in male versus female subjects (1.8% [95% CI = 0.7–3.9] vs. 3.0% [95% CI = 2.4–3.8], respectively, *P* = 0.1) and no significant difference in mean SER between male and female subjects (−0.10 [SD = 2.4] and 0.12 [SD = 2.6], respectively, *P* = 0.26). The higher prevalence of PVD in DZ twins than MZ twins could be explained by a higher average age of DZ twins, given that PVD is strongly age-related.^19^ PVD is a well-established risk factor for retinal detachment and therefore understanding its etiology and factors that influence vitreomacular adhesion is important. GWAS with VMA and PVD may highlight specific genetic loci implicated in these VMI abnormalities. The apparent lower prevalence of PVD in the 80 to 89-year-old group may be that with increasing syneresis with age the posterior vitreous face was anterior to the range of the OCT macular cube and so graded as falsely negative in some participants. Although macular SD-OCT is a valuable tool to visualize posterior vitreous detachment, it may lack sensitivity, especially for detecting complete PVD. The sensitivity and specificity for complete PVD detection on SD-OCT varies between studies, with estimates as low as 37.5% and 31.5%,^38^ up to 71% and 88%, respectively,^39^ whereas the sensitivity of B-scan ultrasonography to detect complete PVD has been reported as 100%.^40^ Despite limitations about the diagnostic accuracy of PVD using SD-OCT on macular but not disc images, we were nevertheless able to show concordance for PVD in both MZ and DZ twin pairs and that PVD (complete or incomplete) is heritable, with a model combining additive genetic and environmental effects providing the best fit.

Our overall prevalence estimate of VMA of 11.8% (95% CI = 10.8–13) is lower than other population-based studies that report using SD-OCT. However, like other population-based studies,^12^^,^^14^^,^^41^ we also showed a decreasing prevalence of VMA with age (after 60 years). This is likely due to complete posterior vitreous detachment which is strongly age-related.^19^

In other studies, VMA has been found to be more common in men than women.^12^^,^^14^^,^^15^^,^^42^ The reasons for this sex difference are unknown; it has been hypothesized that lower levels of hyaluronic acid in women may influence vitreomacular adhesion and lead to earlier posterior vitreous detachment.^43^^,^^44^

The female preponderance of the TwinsUK cohort may partially explain an overall lower population prevalence of VMA, although we found a lower prevalence of VMA in men than women (7.1% [95% CI = 4.9–10.0] vs. 12.5% [95% CI = 11.3–13.7], *P* < 0.001, respectively). Differences in VMA grading between studies and a relatively small proportion of men (13.4%) in TwinsUK may mean we are underpowered to accurately report true sex differences in VMA.

In this twin study, there was a higher proportion of ERM in pseudophakic eyes than phakic eyes which has been reported in previous studies. For example, the Alienor Study (mean age = 79.4 years) reported stage 1 ERM based on SD-OCT imaging in 51% of pseudophakic eyes versus 40.1% in phakic eyes (*P* = 0.0002).^15^ Using the same SD-OCT ERM classification as Alienor, a cross-sectional study of younger participants attending KEYE Eye Centre, Seoul, Korea, for cataract surgery (mean age = 58.7 years) reported an ERM prevalence of 18.2% in phakic eyes.^45^

### Limitations

Although this twin study is large, with a wide age range of subjects all examined with SD-OCT, participants of the TwinsUK cohort are predominantly White Caucasian women and generally healthy. TwinsUK may therefore not be representative of the UK population as a whole and there is likely a healthy volunteer bias. Furthermore, we may be underpowered to accurately quantify sex differences in VMI abnormalities. Participants had no measure of visual acuity and therefore the impact on visual function cannot be assessed. Given the relative rarity of LMH and MH in the population, even with over 3400 participants, we did not have enough power to estimate their heritability. The prevalence of self-reported retinal detachment in this cohort was 1.4%. The estimated incidence of retinal detachment in the United Kingdom is 6.3 to 17.9 cases per 100,000 people.^46^ Questionnaire data are subject to recall bias and it is likely that participants may have confused *retinal* detachment with *posterior vitreous* detachment.

## Conclusion

To our knowledge, this is the first classical twin study to demonstrate the prevalence, concordance, and heritability of common vitreomacular interface abnormalities in a large twin registry with participants from across the United Kingdom. The findings suggest a role for genetic factors in the etiology of VMI abnormalities which warrants further exploration. Consensus is needed to develop an international SD-OCT-based staging system for ERM severity.

## Supplementary Material

Supplement 1
