# Supplementary material for: Prevalence, Concordance, and Heritability of Vitreomacular Interface Abnormalities in a Twin Study
Source: Invest Ophthalmol Vis Sci. 2023 Jul 10;64(10):9. doi: 10.1167/iovs.64.10.9 (PMC10341355; doi:10.1167/iovs.64.10.9)
Supplement: Supplement 1 [file iovs-64-10-9_s001.pdf]

Supplementary Table 1: Percentage agreement and Cohen's Kappa (κ) statistic for each SD-OCT VMI abnormality feature for the entire scan.

**Feature**	**% Agreement**	**Cohen's κ**
ERM	88	0.72
FTMH	100	1
LMH	100	1
PVD	92	0.73
VMA	98	0.92
VMT	100	1

ERM: Epiretinal membrane; FTMH: Full thickness macular hole; LMH: Lamellar macular hole; PVD: Posterior vitreous detachment; VMA: Vitreomacular adhesion; VMT: Vitreomacular traction.
